# Social media impact on public attention: Paris Olympics case study

**DOI:** 10.1371/journal.pone.0336136

**Published:** 2025-11-11

**Authors:** Mingyang Zhang, Yuyue Liao

**Affiliations:** 1 Department of Physical Education, Chengdu University, Chengdu, Sichuan, China; 2 Department of Accounting, Southwest Jiaotong University Hope College, Chengdu, Sichuan, China; Sapienza University of Rome, ITALY

## Abstract

Large-scale sports events yield multi-dimensional positive benefits for urban development, and analyzing their spatiotemporal characteristics is crucial for scientific decision-making. However, existing studies focus on long-term effects, lacking detailed quantitative analysis of short-term dynamics, driving mechanisms, and geographical patterns of attention for single large-scale events using the latest data. Taking the 2024 Paris Olympic Games as a case, this study employs Baidu Index data, hot spot analysis, regression analysis,to explore spatiotemporal characteristics and relational mechanisms of Chinese public and media online attention. Results show: (1) Public and media attention exhibits temporal patterns of pre-event rise, in-event peak, and post-event decline, highly correlated with key competition days; (2) Spatial distribution presents an “east-high, west-low” pattern, with hotspots concentrated in eastern coastal areas; (3) Media attention significantly drives public attention positively, but its influence intensity varies regionally. This study reveals short-term dynamics and spatial differentiation mechanisms of large-scale sports attention in the social media era, providing theoretical and practical references for optimizing communication strategies and enhancing public participation.

## Introduction

Sports events play an important role in modern society, both as an important theme of global cultural exchange and social interaction, and as a potential driving force for economic development. On the one hand, large-scale sports events, as a kind of quasi-public goods, can generate distinct social impacts and benefits; On the other hand, based on the analysis of the attention paid to sports events and the underlying attention patterns, behavioral characteristics, and changing trends, this is helpful in exploring public demand and making scientific industrial decisions, thereby enhancing economic and commercial benefits.

With the popularity and vigorous development of mobile Internet, search engines have become the key entry point for the public to obtain information, providing a new technical environment and data observation window for the formation and evolution of sports attention. In recent years, with the joint promotion of domestic and international modern media such as Facebook, Twitter, TikTok, and Xiaohongshu, various large-scale sports events have been able to achieve dissemination on a wider scale, and the national attention of sports events has also been further enhanced [1]. Among the numerous researches on the attention degree of sports events, it is particularly important to analyze the spatiotemporal characteristics and discuss the changing rules, because it can reflect the public`s attention tendency and attitude preference to sports events under different spatiotemporal conditions.

Through the analysis of the temporal and spatial characteristics behind the attention of sports events, it is often possible to reveal and predict the evolution of the popularity of sports events and its internal relationship with the economic, social and cultural values and behavioral preferences of specific regions. For example, some studies have pointed out that there are periodic fluctuations in the ratings of sports events, and large-scale sports events such as the Beijing Olympic Games are considered to have a significant pulling effect on ratings and attention [2]. Based on Baidu index data during the 2020 Tokyo Olympic Games, some scholars have analyzed that there may be imbalance in sports development level between eastern and western regions of China [3]. In fact, there is evidence that factors such as regional GDP, Internet user size, and the number of athletes participating in the event are closely correlated with the attention of the event.However, although the central role of media in information dissemination has been recognized, the correlation pattern and degree between media coverage intensity and public attention, especially the spatiotemporal dynamic relationship during a specific large-scale event, still need more detailed empirical investigation.

large-scale sports eventslarge-scale sports eventsUndoubtedly, sports events bring positive economic, social, and cultural benefits to urban development, and the analysis of spatiotemporal characteristics and patterns of event attention serves as the premise and basis for scientific decision-making. However, existing research on the attention towards large-scale sports events large-scale sports events remains limited, especially in terms of quantitative analysis of their spatiotemporal characteristics using the latest and large-scale sports event data. As one of the grandest and most influential sports events globally, the 2024 Paris Olympics has reached billions of television viewers and tens of millions of on-site spectators, providing a space for global communication and dialogue. Among them, Chinese audiences have demonstrated their attention and support for international sports events, with sports tourism and event economics providing new impetus and trends for China`s economic development.

This study recognizes the necessity of analyzing the spatiotemporal characteristics of attention towards the Paris Olympics. Specifically, it aims to investigate the temporal and spatial patterns of media and public attention towards Paris Olympic events, and to explore the relationship and interaction mechanism between the two. Based on this, utilizing the Baidu Index, this study employs regression analysis and geographical analysis to uncover the characteristics and patterns of Chinese audience attention towards large-scale sports events. The findings will provide a basis for government and business decision-making, thereby maximizing the economic, social, and cultural benefits of these events.

## Literature review

As an important social and cultural phenomenon, sporting events not only provide people with opportunities for entertainment and leisure, but also have a profound impact on the economy, society, and personal well-being.With the vigorous development of the global sports industry, research on event attention has gradually become a focus of both academia and industry.In recent years, there have been significant changes in the form and mode of dissemination of sporting events.The traditional model of watching games live is deeply integrated with emerging channels such as digital media and social media, creating a multi-dimensional viewing experience [4–7]. Large-scale sports events are the key platform to promote global cultural exchange and economic development. When discussing the attention of large-scale sports events and their influencing factors, many scholars have studied the positive and negative effects of large-scale sports events from the perspectives of economy, society, politics and environment.First, attention to large-scale sports events usually promotes local economic development, including significant increases in tourism revenue, job creation, and stimulating consumption.For example, some studies have revealed the long-term economic contributions of the Olympics to host cities and countries by comparing the economic impact before and after the Olympics [8]. Secondly, the social implications of large-scale sporting events are evident [3]. These events can stimulate community engagement [9], improve the city`s reputation [10] and foster social cohesion [11]. However, they can also exacerbate social disparities [12] and marginalize certain groups [13]. Furthermore, as a medium for cultural communication, sporting events can promote multicultural exchange and inclusive understanding, but they can also trigger cultural conflicts between different ethnic groups and regions [14] and commercialization issues [15]. In terms of environmental and infrastructure impacts, hosting sports events prompts organizers to adopt sustainable practices, such as building green venues or implementing waste management [16], but large sporting events can also lead to short-term environmental pollution and resource consumption [17–19]. In fact, sporting events have a wide-reaching influence, not only demonstrating a country`s soft power and enhancing national pride, but also improving the country`s image on the international stage [20–21]. As mentioned earlier, large-scale sports events are double-edged swords: they can bring economic benefits, but they also come with issues such as high costs and uneven distribution of resources.

In recent years, more and more studies have begun to focus on residents `perception and support during large-scale sports events, analyzing residents` attention, perception changes, perception factors and support during various events [22]. This is because residents` attitudes are crucial to the success of the sports event and the shaping of its influence.With the development of new media, many studies have begun to explore the relationship between social media content producers and the public [23–25], with limited discussion in the fields of media attention and public attention, particularly regarding how media shapes the public`s cognition and attitudes toward specific sports events [26]. In this study, “media attention”refers to the frequency and intensity of news coverage measured by Baidu News Index, while “public attention”refers to user search behavior related to event keywords captured by Baidu Search Index. Empirical research the way media reports, the content of its reports, their accuracy, and the frequency of reporting have a significant impact on residents` behavior and lifestyle [27–28], and the authenticity of reported content facilitates interaction between residents and the media [29]. In fact, the proliferation of new media has expanded the public`s access to event information, resulting in heightened attention towards events. However, this increased attention has introduced complexity. Consequently, future research should prioritize investigating the impact and efficacy of various media in event communication. This entails conducting comprehensive analyses on residents` levels of attention and preferences, with a specific emphasis on factors like temporal and geographical variations,considering how to utilize media and technology to enhance residents` participation and satisfaction, thereby boosting the economic, social, and cultural influence of events. This is conducive to promoting the overall well-being of domestic and international societies through events. The rise of social media platforms has completely transformed the way information is disseminated and consumed, particularly during major sporting events [30]. The Olympics attract attention not only for their sporting events but also for the intense discussions sparked by various issues on social media, spanning topics from environmental concerns to political debates.This literature review aims to explore how public interest in the Olympics is influenced by various factors and how these factors interact with social media to amplify or weaken the event`s impact [31]. Social media has become a key driver of public interest in large-scale sports events. Existing research indicates that social media platforms facilitate real-time discussions, enabling users to share views, emotions, and information related to the event [32]. The Paris Olympics, with its high level of attention, attracted significant online engagement, as evidenced by the numerous hashtags, memes, and discussions surrounding the event [33]. Social media influencers and traditional media institutions played a role in amplifying these discussions, further highlighting the interconnectedness of public attention in the digital age [34].

Multiple factors have been identified as key determinants of public attention during major sporting events. The nature of the event itself, such as its scale, significance, and novelty, plays a crucial role [35]. As a quadrennial event with a long history, the Paris Olympics naturally attract global attention. However, other factors, such as event organization, media coverage, and the social and political context, also significantly influence public engagement. For example, environmental concerns arising from water quality issues in the Seine River during the Paris Olympics sparked widespread discussion on social media [36]. The French government`s efforts to address these issues, including the mayor`s symbolic swim in the river, generated extensive discussion, highlighting the complex interaction between public perception and government action. Similarly, the event`s organizational work, including issues such as accommodation quality and catering services, also drew widespread attention and criticism on social media [37]. The Paris Olympics provide a vivid example of how public attention in the social media era is influenced by various factors. The high level of public attention combined with the widespread use of social media has facilitated real-time discussions and debates [38]. However, it is the interaction between event organization, media coverage, and the socio-political context that truly determines the nature and extent of public attention [39]. For example, the controversy surrounding the water quality of the Seine River not only raised environmental concerns but also sparked debates about the sustainability of the event and the government`s commitment to addressing such issues [40]. Similarly, criticism of the event`s accommodation and catering services highlighted the importance of event organization in shaping public perception [41]. In summary, the case study of the Paris Olympics reveals the complex dynamics of public attention in the social media era. Existing research has primarily focused on the long-term positive and negative impacts of major sporting events on host cities and nations, with limited attention given to the short-term dynamics observable within the timeframe of a single Olympic Games. Additionally, there has been insufficient exploration of the real-time evolution of public attention, its driving mechanisms, and geographical patterns in the social media era. Existing research primarily relies on traditional statistical data or post-event surveys, making it difficult to capture real-time fluctuations in public attention during the event and even more challenging to reveal the dynamic interplay between information dissemination, user interaction, and changes in public attention on social media platforms. As a major global sporting event in the post-pandemic era, the Paris Olympics coincided with a critical phase of social media`s deep penetration into public life. The event`s high level of attention, widespread social media usage, and various influencing factors—such as event organization, media coverage, and socio-political context—played a key role in shaping public engagement [42], providing a typical case study for researching the formation and evolution of public attention in the social media era. Therefore, this study takes the Paris Olympics as its entry point, focusing on the dynamics of public attention within the timeframe of a single event. This not only fills the gap in existing research on short-term attention analysis but also reveals the unique patterns of attention toward major sporting events in the social media environment, providing theoretical foundations and practical references for event organizers to optimize communication strategies and enhance public participation.

## Research design

### Research subjects

**Fig 1:**
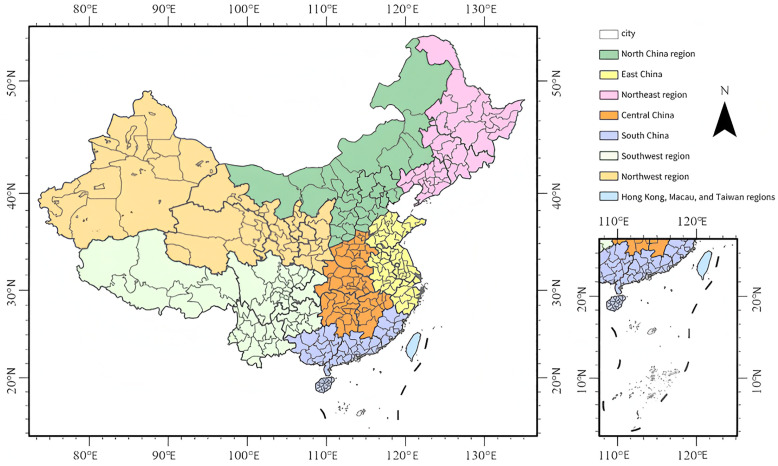
Map of the study area. (The map was created by the authors using ArcGIS Pro. The base map was used under the official standard map review number GS(2024)0650, in compliance with Chinese cartographic regulations).

To analyze the attention towards large-scale sports events and its influencing factors, this study selects the 2024 Paris Olympic Games as the research subject. The reason for this choice is that, as a top-tier global sporting event, it not only brings together the competitive prowess of top athletes from around the world but also serves as a focal point of extensive media and public attention both domestically and internationally. A deep understanding of the trends in media and public hotspots during large-scale sports events can help analyze the underlying temporal and spatial impacts and relational mechanisms behind them.Baidu Index reflects the search behavior of Chinese internet users (Fig 1).

### Data sources

The data for this study were compiled from three primary sources: public web search data, media information data, and macroeconomic social data. The core dataset was sourced from the Baidu Index platform. Baidu, China`s dominant search engine (analogous to Google in Western markets), processes over 70% of domestic search queries. Its Baidu Index tool aggregates anonymized search data, offering a robust proxy for real-time public interest and attention. This study leverages this platform to ensure the data`s authority and representativeness.

To precisely capture the dynamics of public attention, this study utilizes the Baidu Search Index as its core analytical tool. The data collection window was set from July 19, 2024, to August 18, 2024. We selected “Paris Olympics” as the primary overview keyword, supplemented by a series of specific terms such as “Olympics live broadcast”, “Olympics medal tally”, “Olympics opening ceremony”, and “Olympics schedule”. This multi-keyword approach allows for a comprehensive tracking of public interest across different facets of the event, from both macro and micro perspectives.

The quantitative analysis of media attention relies on the Baidu News Index. By retrieving the index for the core keyword “Paris Olympics” within the same timeframe (July 19 to August 18, 2024), we obtained daily data on the frequency of news reports and the intensity of public opinion across mainstream online media. This metric is crucial for gauging the strength of the media`s agenda-setting function and analyzing its association with public attention, ensuring that media coverage and public response are temporally aligned and comparable.

Furthermore, to control for potential confounding effects arising from regional socioeconomic disparities, we incorporated macroeconomic statistical data as control variables. Sourced from the latest Statistical Yearbooks published by national and provincial/municipal bureaus of statistics, this study selected resident population and urbanization rate as key indicators. The inclusion of these variables is intended to isolate the exogenous influence of population size and regional development levels in the model analysis, thereby allowing for a more focused examination of the relationship between media coverage and public attention.

### Research methods

In exploring the temporal and spatial characteristics and relationship mechanisms of indicators, this study employs statistical methods including hot spot analysis, regression analysis, and MGWR (Multiscale Geographically Weighted Regression) analysis.

#### Optimized hot spot analysis.

Optimized hot spot analysis. The Getis-Ord Gi* statistic is the core mathematical tool in hot spot analysis, used to identify patterns of high or low value clustering in spatial data. Below is a brief explanation of its mathematical principles. Among them, local spatial autocorrelation is measured by the Getis-Ord Gi* statistic, which evaluates the correlation between the values in one area and those in its neighboring areas. It is used to detect clusters of abnormally high values (hot spots) or low values (cold spots) in spatial data.

#### Generalized linear regression.

Generalized Linear Regression (GLR) is an extension of linear regression that allows the dependent variable to follow a distribution within the exponential family, with a link function connecting the linear predictor to the mean of the dependent variable. The following represents the research methodology of generalized linear regression [43,44].


$$g\left(E\left(Y\right)\right)=\beta_{0}+\beta_{1}X_{1}+\beta_{2}X_{2}+\ldots +\beta_{p}X_{p}$$ (1)

In this context, Y represents the dependent variable, E(Y) is the expected value of the dependent variable, g(·) is the link function, $\beta_{\text{0}}\text{,}\beta_{\text{1}}\text{,}\ldots \text{,}\beta_{p}$ represents the model parameters, and $X_{\text{0}}\text{,}X_{\text{1}}\text{,}\ldots \text{,}X_{p}$ stands for the independent variables.

#### Multi-scale geographically weighted regression.

The Multi-scale Geographically Weighted Regression (MGWR) model is a spatial model used to explore the geographical variation in relationships between dependent/response variables and independent/explanatory variables. Building upon the classic GWR model, the MGWR model incorporates spatially stationary variables, allowing each influencing factor to have its unique bandwidth value, thereby rendering the model estimates more realistic and reliable [45].


$$y_{i}=\sum_{k=1}^{m}\alpha_{k}X_{ik}+\sum_{k=n+1}^{n}\beta_{k}(u_{i},v_{i})x_{ik}+\varepsilon_{i}$$ (2)

In Equation (2): k represents the number of influencing factors, $\alpha_{k}$ denotes the global variable regression coefficient, $\beta_{k}$ represents the local variable regression coefficients, $X_{ik}$ is the value of the k th influencing factor at the i th location, $\left(u_{i},v_{i}\right)$ represents the spatial coordinates of the i th element, $\varepsilon_{i}$ represents the residual error.

#### Multivariate cluster analysis.

This paper uses the Multivariate Clustering analysis method in ArcGIS Pro to explore the spatial distribution characteristics of public attention in different cities during the Paris Olympic Games by analyzing the influence coefficients of media attention, residents` attention, and media attention on residents` attention in 337 prefecture-level and above administrative units across the country. Cluster analysis uses K-Means algorithm combined with Akaike Information Criterion (AIC) to optimize the number of clusters, classifies cities with high similarity through an iterative process, and reveals the potential patterns of cities in media attention and residents` response. This paper avoids the interference of dimensional differences on the clustering results by standardizing the relevant variables to ensure the reliability and consistency of the analysis results.


$$AICc=2k-2\ln\left(L\right)+\frac{2k\left(k+1\right)}{n-k-1}$$ (3)

Where, k is the number of estimated parameters in the model (such as the number of clusters), L is the value of the likelihood function corresponding to the maximum likelihood estimation, n is the total number of samples, $\frac{2k\left(k+1\right)}{n-k-1}$ the small sample correction term of AIC.


$$\begin{array}{c} \min{\mathrm{\backslash nolimits}}_{C}\sum {\mathrm{\backslash nolimits}}_{k=1}^{K}{\sum {\mathrm{\backslash nolimits}}_{x_{i}\in C_{k}}\left\|x_{i}-\mu_{k}\right\|}^{2} \end{array}$$ (4)

In Equation (4), C is the set of all clusters, where K is the total number of clusters and $C_{k}$ is the $k^{th}$ cluster, The term $x_{i}$ denotes a sample point, and $\mu_{k}$ represents the centroid of the $k^{th}$ cluster, defined as the mean of all points within that cluster. ${\left\|x_{i}-\mu_{k}\right\|}^{2}$ is the squared Euclidean distance between a sample point $x_{i}$ and the centroid $\mu_{k}$ of the cluster to which it belongs.

## Analysis of results

### Temporal and spatial characteristics of public attention

Based on Baidu Search Index, daily search volumes for keywords such as “Paris Olympic Games,” “Olympic Games live broadcast,” “Olympic Games medal tally,” “Olympic Games opening ceremony,” “Olympic Games closing ceremony,” “Olympic Games schedule,” “Olympic Games gold medals,” and “Olympic Games playback” were analyzed between July 19,2024 and August 18, 2024.Temporal evolution characteristics

**Fig 2:**
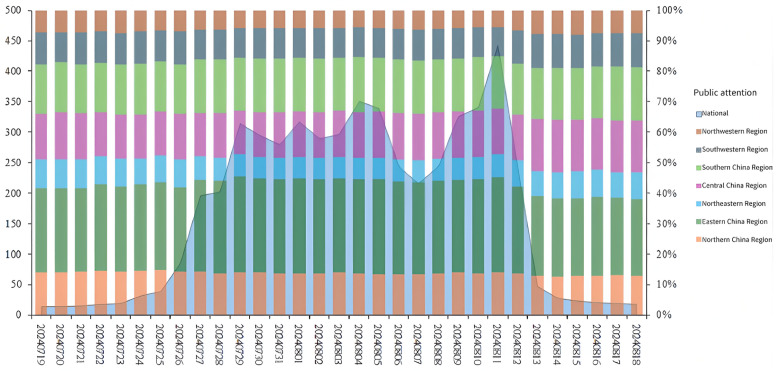
Public attention. **Note:**The temporal and spatial evolution of public attention for the Paris Olympics (July 19 to August 18, 2024).Public attention surged sharply after the opening ceremony on July 26, remained at a high level during the competition period, and dropped sharply after the closing ceremony on August 11. It reached its peak on August 11 (the last day of competition), when China achieved its best performance at an overseas Olympics. Secondary peaks on July 29, August 1–5, and August 9 were associated with key victories by the Chinese team and performances by star athletes, particularly in table tennis and swimming.

As shown in Fig 2, public attention rose rapidly after the opening ceremony on July 26th and maintained a high level during the competition days. Following the closing ceremony on August 11th, attention declined sharply. A peak in attention occurred on August 11th, which was the final competition day of the Paris Olympic Games. On this day, weightlifter Li Wenwen won the 40th gold medal for China, marking the best performance of the Chinese delegation at an Olympic Games held abroad. The final results of the Olympics also garnered intense public attention, forming a peak in attention. Similar to media attention, public attention declined rapidly with the conclusion of the Olympics.

On July 29th, August 1st, 4th, 5th, and 9th, the public attention reached secondary peaks. On July 29th, the Chinese table tennis team advanced to the finals in the mixed doubles semifinals, and at the same time, the Chinese women`s volleyball team faced and defeated the powerful United States team in their first match, attracting widespread attention. On August 1st, the Chinese men`s three-on-three basketball team surprisingly defeated the previous champions, Serbia. Additionally, Pan Zhanle, competing for the first time, won the first gold medal for the Chinese swimming team at this Olympics. On the same day, the table tennis singles quarterfinals also took place consecutively, drawing the public`s attention. During the secondary peaks on August 4th and 5th, the Chinese delegation won a total of five gold medals. Among them, Fan Zhendong defeated his opponent to win the men`s singles title in table tennis, while Pan Zhanle contributed to China`s gold medal in the men`s 4x100m medley relay with a final-leg reversal. Furthermore, gymnasts Liu Yang and Zou Jingyuan successfully defended their titles. These key events significantly boosted public attention. Starting from August 8th, public attention rose once again and peaked on August 11th. Between August 8th and 10th, China won a total of 14 gold medals, and the continuous growth in the number of gold medals attracted significant public attention. As shown in the graph, the public paid closer attention to the key events of the Chinese team and the competitions involving star athletes, especially showing a high level of interest in the Chinese table tennis team.

Upon examining the distribution of attention across China`s seven major economic regions in detail, it is found that the eastern China region accounts for the highest proportion of attention, with a notable increase during the competition days. The proportions of attention in northern China, central China, and southern China are similar, while the northwestern and northeastern regions have the lowest proportions of attention.

#### Spatial distribution characteristics.

**Fig 3:**
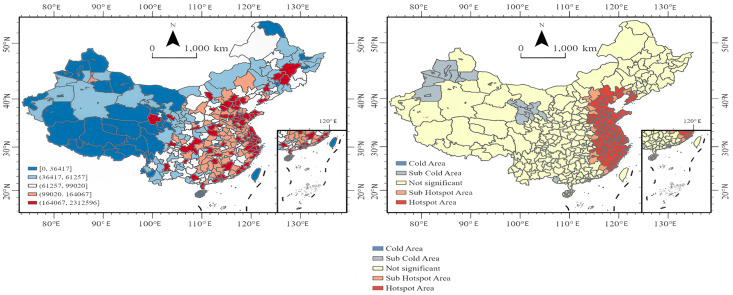
Public attention. **Note:**Spatial distribution of public attention toward the Paris Olympics (July 19 to August 18, 2024).Left figure: Spatial distribution of public attention, with high values concentrated in eastern coastal cities. Key central and western provincial capitals (such as Hohhot, Lanzhou, Chengdu, and Chongqing) also exhibit relatively high levels of attention, consistent with the distribution of media attention in regional core cities.Right figure: Hotspot analysis results. Red areas indicate public attention hotspots, primarily distributed in eastern, northern, and northeastern coastal cities. (The map was created by the authors using ArcGIS Pro. The base map was used under the official standard map review number GS(2024)0650, in compliance with Chinese cartographic regulations).

Fig 3 presents the spatial distribution of public attention. The left graph reveals that the public in eastern coastal cities demonstrates higher levels of attention, while attention in western regions is relatively low. Only cities such as Hohhot, Lanzhou, Chengdu, and Chongqing show higher levels of attention, similar to the distribution of media attention, which focuses on capital cities in central and western regions. The hot spot map on the right indicates that hot spots are mainly located in coastal cities in East China, North China, and Northeast China. Specifically, Dalian, Anshan, Yingkou, Huludao, and Chaoyang in Northeast China are hot spots for public attention, all of which are cities along the Bohai Bay. There are no cold spots for public attention nationwide; only some areas in Xinjiang Uyghur Autonomous Region and Gansu Province have secondary cold spots.

### Temporal and spatial characteristics of media attention

#### Temporal evolution characteristics.

**Fig 4:**
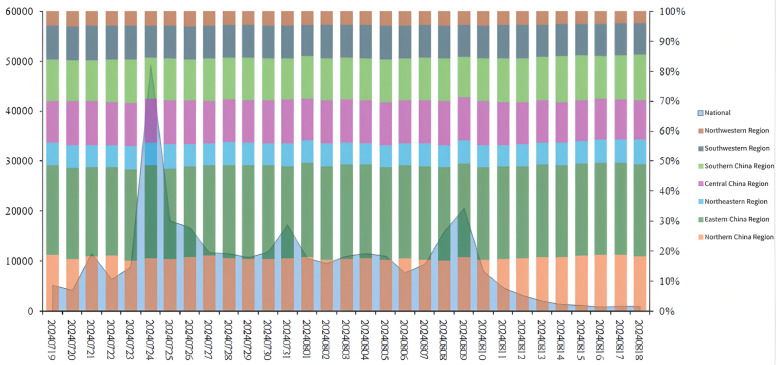
Media attention. **Note:**Temporal evolution of media attention to the Paris Olympics (July 19–August 18, 2024).Media attention peaked on July 24. Secondary peaks on July 21, 26, 31, and August 9 aligned with pre-event preparations, the opening ceremony, and key Chinese athlete gold medal wins. Attention remained high during table tennis events and declined rapidly post-closing ceremony.

As shown in Fig 4, the peak of the Media Information Index occurred on July 24th, when the Chinese Olympic delegation for the Paris Olympics announced that the flag bearers for the opening ceremony would be male table tennis player Ma Long and female synchronized swimmer Feng Yu. Both are captains of their respective team sports, and this is Ma Long`s fourth Olympic Games. As a veteran of the national table tennis team, he not only garners attention from domestic media but also receives significant attention from the International Olympic Committee. On the 24th, the official competition of the Paris Olympics kicked off with rugby, handball, and soccer matches leading the way. This day also marked the official start of the Paris Olympics. Therefore, the Media Information Index reached a noticeable peak on the 24th, reflecting the intense attention of domestic media towards the Olympics and related news about the national team.

Secondly, secondary peaks were also observed on July 21st, July 26th, July 31st, and August 9th, which were closely related to China`s championship victories in relevant events on those days. On the 21st, the Paris Olympic Organizing Committee held a press conference on preparations, through which Chinese media learned about and reported on the Olympic preparations. On the 26th, the Paris Olympics held its opening ceremony on the Seine River, marking the first time the opening ceremony was held outside the main stadium. Therefore, Chinese media provided continuous coverage of the opening ceremony on the 26th. During the competition day on July 31st, Chinese athletes won a total of three gold medals. Among them, the highly anticipated diving duo Quan Hongchan and Chen Yuxi secured the gold medal in the women`s synchronized 10m platform event, successfully defending their title since the Tokyo Olympics. Additionally, Deng Yawen won China`s first gold medal in the freestyle BMX competition. Furthermore, the national men`s and women`s 3x3 basketball teams, as well as the women`s basketball team, each made their debut at the Paris Olympics. On July 30th, August 3rd, 4th, as well as August 9th and 10th, media attention was at a relatively high level, coinciding with the times when gold medals were won in table tennis events, reflecting the media`s greater focus on table tennis competitions. With the conclusion of the closing ceremony, media attention rapidly declined.

Upon closely examining the attention distribution across China`s seven major economic regions, it can be observed that the eastern China region accounts for the highest proportion. The combined proportion of eastern and northern China regions is close to 50%. The northwestern region has the lowest level of attention, which continues to decline as the competition progresses. Changes in other regions are not significant.

#### Spatial distribution characteristics.

**Fig 5:**
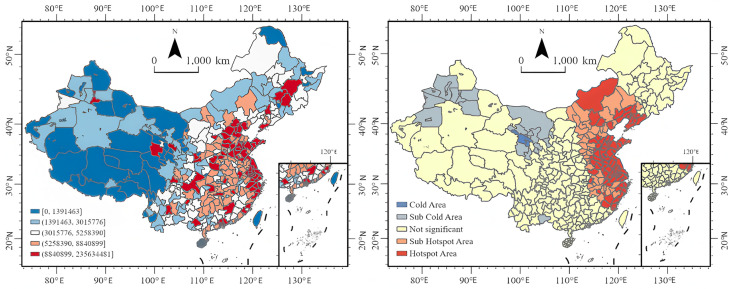
Media attention. **Note:**Spatial distribution of media attention to the Paris Olympics (July 19–August 18, 2024).Left: Media attention is higher in eastern coastal regions, with key western provincial capitalsalso showing elevated levels due to richer media resources.Right: Hotspot analysis reveals most eastern China, parts of northern and central China as media attention hotspots; cold spots are limited to Haibei Tibetan Autonomous Prefecture and Xining. (The map was created by the authors using ArcGIS Pro. The base map was used under the official standard map review number GS(2024)0650, in compliance with Chinese cartographic regulations).

According to Fig 5, the left graph shows that media attention in the eastern coastal regions is relatively high, while that in the western regions is lower. However, certain cities in the western region, such as Urumqi, Lanzhou, Chengdu, and Kunming, which are the capital cities of their respective provinces or autonomous regions, exhibit higher media attention. Compared to other cities within the same province or autonomous region, these cities have better economic development, larger population distributions, and a richer variety of media. The cold spot distribution map on the right shows that the entire eastern China region, most of the northern China region, and a small part of the central China region have higher media attention hotspots. The majority of cities in the eastern China region are hotspots, while other cities are secondary hotspots. Some cities in the northern China region are hotspots, and a few cities in the northeastern and central China regions are also hotspots. The northwestern and southwestern regions have insignificant media attention or are cold spot areas, with Haibei Tibetan Autonomous Prefecture and Xining being the only two cold spot areas nationwide.

From Figs 2 and 4, it can be observed that the media pays higher attention to matches featuring star athletes, critical matchups, and competition days with a higher number of gold medals awarded. Meanwhile, the public maintains a high level of attention on competition days but shows significantly less interest on non-competition days. Both media and public attention are highest in the eastern China region and lowest in the northwestern region. As Figs 3 and 5 illustrate, the high-value areas for both media and public attention are roughly similar, concentrated in eastern coastal cities, while western regions show lower levels of attention. However, some provincial capitals and autonomous region capitals in the west have higher levels of attention. The eastern China region is a hotspot for both media and public attention. It is evident that areas with developed economies and larger populations have higher levels of attention.

### Analysis of the influence of media attention on public attention

#### Overall perspective.

**Fig 6:**
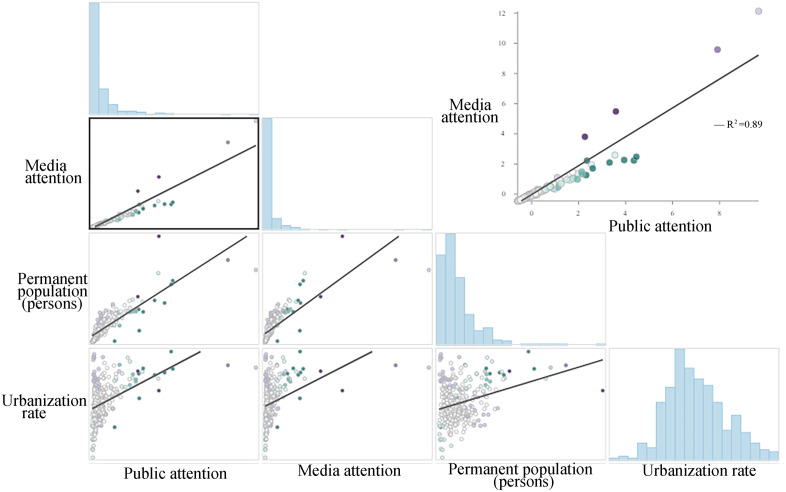
Correlation between permanent resident population, urbanization rate, media attention, and residents` attention. **Note:**Correlation between media attention, resident population, urbanization rate, and public attention.Scatter plots show a strong positive linear correlation between media attention and public attention (R² = 0.89), while correlations with resident population and urbanization rate are weaker.

To systematically investigate the core role of media coverage in shaping public attention towards major sporting events, this study conducted a comprehensive national-level analysis of key metrics, including media attention, resident attention, resident population, and urbanization rate. The primary finding reveals an exceptionally strong positive linear relationship between media attention and resident attention (Fig 6). Scatter plot analysis clearly shows that data points for various cities are highly clustered around the regression line, with a calculated coefficient of determination (R^2^) of 0.89. This compelling figure indicates that media attention can explain nearly 90% of the variance in resident attention, suggesting that the intensity and breadth of media coverage are the dominant predictors of public interest and online search behavior. This finding provides powerful evidence for the classic “Agenda-Setting” theory in the digital age, demonstrating that the media agenda largely guides and shapes the public`s focus of attention.

In contrast, the influence of socio-structural factors such as resident population and urbanization rate is significantly weaker. The analysis shows that the correlation of these two variables with resident attention is far less tight than that of media attention, with their data points appearing more dispersed on the plot. This suggests that a large population merely provides a potential user base for high attention, and a high urbanization rate only represents a favorable environment for information dissemination; neither, on its own, is a direct or sufficient condition for activating public attention for a short-term event. These factors act more as “slow variables” that influence public information behavior by providing a foundational social context, but they are far less immediate and decisive than media promotion in shaping collective attention on specific, short-term events.

Based on the stark contrasts revealed in the correlation analysis, this study constructs a Generalized Linear Model (GLM) wherein media attention is set as the core explanatory variable, while resident population and urbanization rate serve as control variables. The objective of this model design is to isolate the net effect of media coverage by statistically controlling for pre-existing conditions such as city size and development level. Through this approach, we aim to precisely assess the independent impact of media attention on resident attention, thereby providing robust evidence that, during the Paris Olympics, media coverage was the most crucial and direct catalyst for sparking and guiding the surge in public interest.

The GLR model fitting diagnostic information (Table 1) yields an R^2^ of 0.93, which is relatively high, indicating that the three factors of media attention, urbanization rate, and permanent resident population have a high degree of explanatory power for residents` attention. Additionally, the generalized linear regression (GLM) model constructed in this study obtained a lower modified Akaike Information Criterion (AIC_C)_ value, which was significantly lower than the ordinary least squares (OLS) model used as a reference (AICC = 83.49). This indicates that GLM is a better choice after considering the goodness of fit and parameter complexity of the model, and its analysis results have higher statistical validity.

**Table 1:** Diagnostic information for GLR model fitting.

Dependent variable	core variable	Control variable	R^2^	After correction R^2^	AIC_C_
Public attention	Media attention	Permanent population, Urbanization rate	0.93	0.93	75.56

Based on the analysis results of the Generalized Linear Regression (GLR) model, we can observe that media attention has a significant positive impact on residents` attention. Specifically, the coefficient of media attention is 0.69, which indicates that as media attention increases, residents` attention to sporting events also increases correspondingly (Table 2). This finding highlights the important role of media in shaping public interests and behaviors, especially in major sporting events such as the Olympics and other international competitions. Therefore, it can be concluded that increasing media attention not only enhances the visibility of sporting events but also significantly boosts public participation and interest.

**Table 2:** Results of GLR model fitting.

Variable	Coefficient	Standard deviation	Significance	VIF
Media Attention	0.69	0.02	0.00	2.80

**Fig 7:**
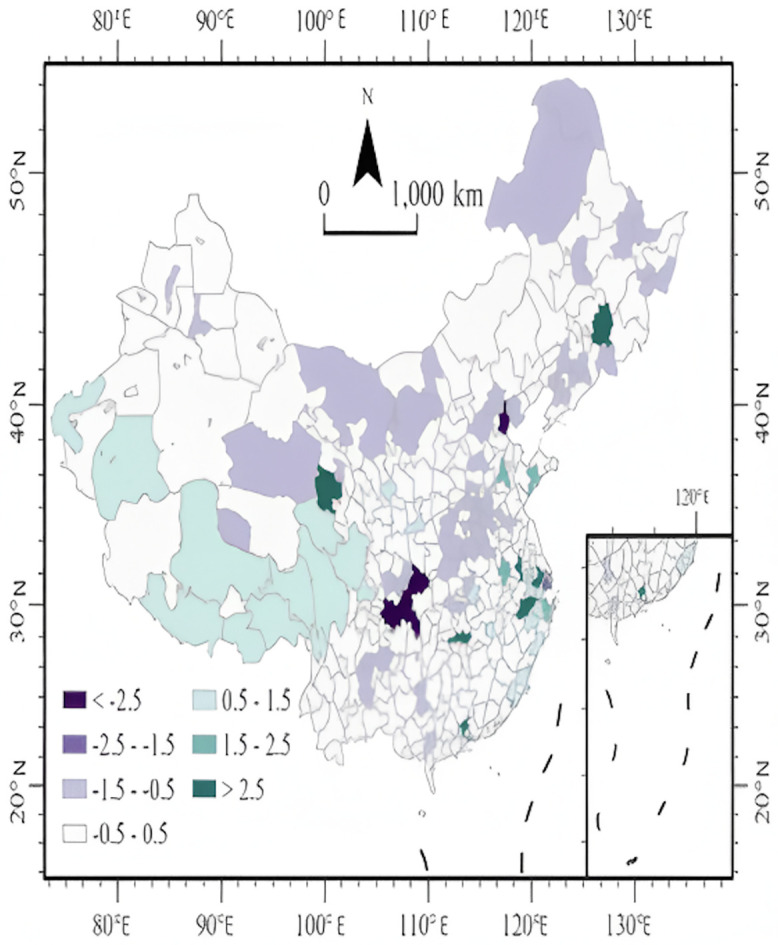
Spatial distribution map of standardized residuals. **Note:**Regions with high positive residuals show public attention exceeding model predictions, linked to developed sports cultures. Negative residuals in northeastern, northwestern, and parts of central China reflect lower-than-predicted attention. (The map was created by the authors using ArcGIS Pro. The base map was used under the official standard map review number GS(2024)0650, in compliance with Chinese cartographic regulations.)Local perspective.

An analysis of the model`s residuals, as illustrated in Fig 7, reveals distinct geographical patterns. Regions with high positive standardized residuals—indicating that actual attention was significantly higher than predicted by the model—are predominantly concentrated in eastern coastal areas and several economically advanced cities, such as Suzhou, Hangzhou, Chongqing, and Tianjin. These cities are not only characterized by rapid economic development and rapid information dissemination, but also have a good sports culture atmosphere, and the local people have a certain level of sports awareness [46–49]. Consequently, their level of attention surpassed the forecasts based on media and demographic data alone. The case of Hangzhou is particularly illustrative, with a standardized residual of +2.3. This signifies that its actual resident attention was approximately 2.3 standard deviations above the predicted value, The case of Hangzhou is particularly typical, with a standardized residual of +2.3. This indicates that the actual resident interest is approximately 2.3 standard deviations higher than the predicted value. This suggests the influence of factors not considered by the model, such as a deep-rooted local sports tradition, unique regional culture, or experience in hosting large-scale events [50]..Conversely, areas marked by significant negative residuals are primarily located in the three northeastern provinces, the northwestern region, and parts of central China, including cities like Jilin and areas within Qinghai. In these regions, resident attention fell markedly below the model`s predictions, with residuals commonly ranging from −1.5 to −2.1. This discrepancy may reflect regional challenges, such as deficiencies in media information reception, a comparatively lower level of public interest in national sporting events, or less developed social support systems for such activities,the situation in parts of Qinghai is especially telling; despite considerable media attention directed at the region, resident attention did not rise commensurately. This points to potential structural issues, such as ineffective communication strategies or media content that lacks local relevance and fails to resonate with the regional population [51].

**Fig 8:**
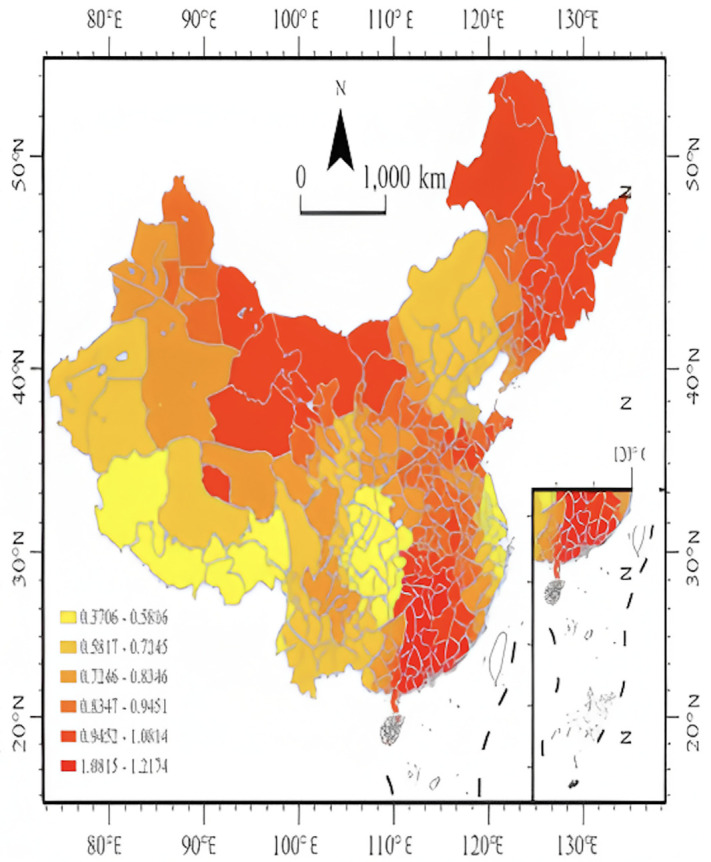
MGWR regression coefficient map of media attention on residents attention. **Note:**Media influence is stronger in northeastern, northwestern, and central-southern regions, where residents rely more on media. Lower coefficients in eastern coastal areas reflect diversified information sources reducing media dependence. (The map was created by the authors using ArcGIS Pro. The base map was used under the official standard map review number GS(2024)0650, in compliance with Chinese cartographic regulations.).

According to the data in Fig 8, the impact coefficient of media attention on residents` attention is positive across the country, indicating that media reporting has a significant positive effect on enhancing residents` attention to sporting events in all regions. From a spatial distribution perspective, the high-value areas of the impact coefficient are mainly concentrated in the Northeast, Northwest, and some central and southern regions, such as Heilongjiang Province, Jilin Province, Liaoning Province, Guangdong Province, Hunan Province, Jiangxi Province, Qinghai Province, and Xinjiang Uyghur Autonomous Region. The relatively high impact coefficients in these areas are closely related to the relatively concentrated information dissemination channels and the leading role of traditional media in reporting on sporting events. Especially in situations where information sources are limited, residents have a higher dependence on media reports. In contrast, the impact coefficients in developed eastern coastal regions such as Jiangsu Province, Zhejiang Province, and Shanghai are relatively low. In these areas, where sports culture and industry are mature, residents` attention to sporting events stems more from personal long-term interests and participation experiences rather than relying on media reports. Additionally, the influence of social circles on event attention is greater. Through information diffusion via social networks, residents are more susceptible to the influence of friends, colleagues, and others, further weakening the direct effect of media on attention [52].

Overall, the guiding role of media in shaping residents` attention to sporting events is more pronounced in less developed regions. However, in economically developed areas, attention to sporting events is influenced by multiple information sources, and the role of media is relatively diminished. This phenomenon reflects significant differences in residents` dependence on media for accessing sporting event information across different regions, warranting further cluster analysis.

**Fig 9:**
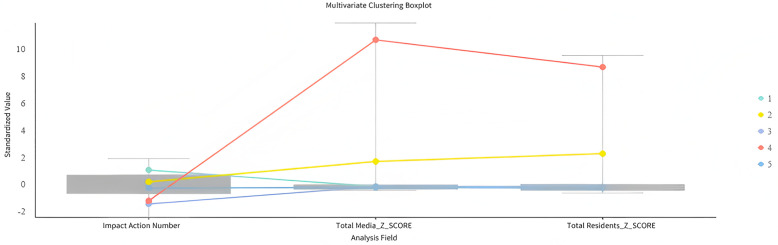
Box plot of multivariate clustering. **Note:**Cities are categorized into five types based on media attention, public attention, and media impact coefficients. Type 1 shows the strongest media influence, while Type 4 has high attention but low media impact.

The study conducted a multivariate cluster analysis on all cities based on media attention, residents` attention, and the impact coefficient of media on residents` attention. According to the results of the box plot (Fig 9), all cities can be divided into five categories, with an R^2^ value of 96.33%, indicating a scientific classification. Specifically, the media impact coefficient of the first category of cities is significantly higher than that of other categories. Although their media attention and residents` attention are not the highest, the guiding role of media in residents` attention is the most significant, suggesting that residents in these cities have a high dependence on media reports, and the role of media in public opinion guidance is particularly prominent. In contrast, the media impact coefficients, media attention, and residents` attention in the second, third, and fifth categories of cities are all at relatively low levels, indicating that media reports on sporting events in these cities are scarce, and their guiding role in residents` attention is weak. It is noteworthy that the standardized values of media attention and residents` attention in the fourth category of cities are the highest, indicating that the frequency of media reports on sporting events and residents` attention in these cities are relatively prominent. However, the impact coefficient of media on residents` attention in these cities is relatively low, reflecting a limited direct guiding role of media. Residents in these cities may rely on diversified information acquisition channels, such as social media and offline social circles. Overall, there is not a simple linear relationship between the frequency of media reports and their impact on residents` attention to sporting events. Instead, it is jointly influenced by the regional media environment, diversity of information channels, and residents` sports culture foundation.

**Fig 10:**
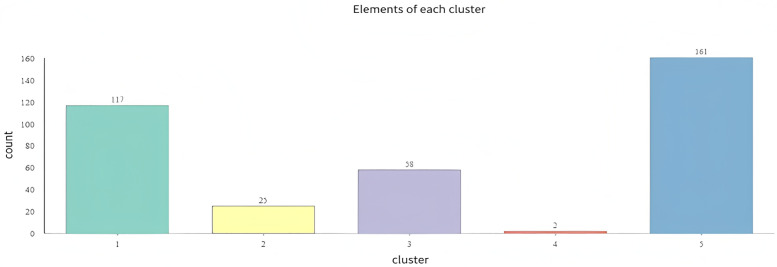
Statistical chart of city classification by quantity. **Note**:Type 5 cities are most numerous, followed by Type 1, indicating prevalent low-attention or high-media-influence characteristics. Types 2 and 4 are rare, reflecting unique attention patterns in specific regions.

Statistical results on the number of cities across five categories reveal significant differences in the distribution of cities among various categories (Fig 10). Cities of the fifth type are the most numerous, totaling 161, accounting for over 75% of the classification along with cities of the first type. This indicates that the characteristics of these two types of cities are the most prevalent across multiple dimensions. There are 58 cities of the third type and only 25 cities of the second type, suggesting that these types of cities are relatively uncommon. Cities of the fourth type are the least numerous, encompassing only 2 cities, which demonstrates that the characteristics of this type of city are the rarest among all cities. This distribution reflects variations in city characteristics across multiple dimensions, such as media attention, resident attention, and media influence coefficients, further illustrating the apparent imbalance in city classification.

The spatial distribution display of the classification of all cities nationwide clearly reveals the geographical and spatial differences in characteristics among various types of cities. Spatially, cities in the eastern coastal regions exhibit a more diversified type distribution, attributed to their developed economies, dense populations, and more diversified information dissemination channels. In contrast, cities of the fifth type dominate in the western regions, reflecting the relatively low media and resident attention in these areas, which is associated with factors such as lower economic development levels and limited information dissemination channels. Cities of the third type are relatively concentrated in the central and southwestern regions, indicating that these areas are in a transitional state in terms of media dissemination and resident attention. Although they are less diverse than the developed eastern regions, they are more diverse than the western regions.

**Fig 11:**
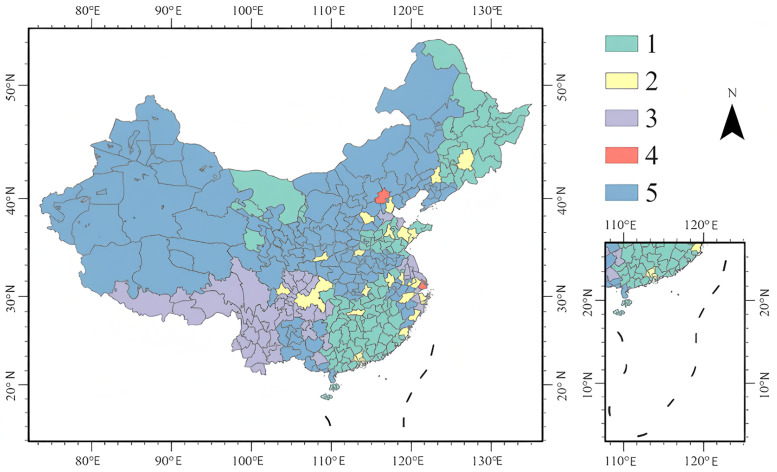
Distribution of city types based on multivariate clustering. **Note:**Eastern coastal cities show diverse types due to developed information channels. Western regions are dominated by Type 5. Type 1 clusters in northeastern, northern, and southwestern China; Type 4 is limited to Beijing and Shanghai. (The map was created by the authors using ArcGIS Pro. The base map was used under the official standard map review number GS(2024)0650, in compliance with Chinese cartographic regulations).

As shown in Fig 11, cities of the first type are mainly distributed in Northeast China, North China, and Southwest China. These cities have high media and resident attention, with the media playing a significant guiding role. Cities of the second type exhibit a relatively dispersed distribution pattern, concentrated in the more developed cities of the central and eastern provinces. The number of such cities is relatively small, indicating that the attention characteristics of this type of city are relatively unique. Cities of the third type are distributed in central and southwestern regions, especially in Sichuan, Hubei, Hunan, and other places, demonstrating moderate media influence and resident attention. Cities of the fourth type are the least numerous, occurring only in Beijing and Shanghai, both of which are municipalities directly under the central government in China. Many domestic and foreign media outlets are stationed here, resulting in a large scale of media information. Both Beijing and Shanghai possess a profound sports culture and a vast group of sports fans, with residents showing a high interest and enthusiasm for sports. However, the influence of media and resident attention in these two cities is low.. Cities of the fifth type are widely distributed in northwest, north, and south China, showing obvious ubiquity. Their media and resident attention are relatively balanced but have overall weak influence.

## Conclusions and discussion

This study takes the 2024 Paris Olympic Games as its research object. Using Baidu Index data and methods such as hotspot analysis, regression analysis, and multi-scale geographically weighted regression (MGWR), it systematically explores the spatiotemporal characteristics of public and media network attention in large-scale sporting events and the relationship mechanism between the two.

Public and media attention toward the Paris Olympics exhibits a distinct pattern of phased changes. Public interest surged rapidly after the opening ceremony, remained at a high level during the games, and dropped sharply after the closing ceremony. Notable peaks were observed on key competition days, such as the final day when the Chinese delegation achieved its best-ever overseas Olympic performance and important match days when Chinese athletes won gold medals; Media attention reached peaks at key moments such as the announcement of the flag bearer for the delegation, the official start of the events, and Chinese athletes winning gold medals. In particular, the concentrated reporting period for popular events like table tennis closely aligned with the peaks in media attention. The fluctuations in both public and media attention were closely tied to key milestones in the course of the events.The spatial distribution of public and media attention exhibits an “east-high, west-low” pattern. The eastern coastal regions are areas of high attention, while the western regions have relatively lower attention; however, some central and western provincial capitals (such as Chengdu, Chongqing, and Lanzhou) exhibit high attention due to their strong economic foundations and abundant media resources. Public attention hotspots are concentrated in eastern China, northern China, and northeastern coastal cities (especially the Bohai Bay region), while media attention hotspots are primarily distributed across most of eastern China, northern China, and parts of central China.Media attention has a significant positive driving effect on public attention, with the two exhibiting a strong linear correlation (R² = 0.89), thereby validating the applicability of the “agenda-setting” theory in the digital age. In contrast, the influence of social structural factors such as permanent population and urbanization rates is relatively weak. Additionally, this driving effect exhibits regional heterogeneity: in northeastern, northwestern, and certain central-southern regions where information dissemination channels are relatively concentrated, the media`s guiding role in shaping public attention is more pronounced. However, in economically developed regions such as the eastern coastal areas, where the public relies on diverse information sources and is more influenced by personal interests and social networks, the media`s direct impact is relatively weaker.

This study employs an empirical analysis of the Paris Olympics to reveal the dynamic patterns and influencing mechanisms of public and media attention in major sporting events from both temporal and spatial dimensions, offering a new perspective on the complex formation process of event attention.

The finding that media coverage directly influences public attention to sporting events by shaping public perceptions and preferences aligns with Katz et al.`s “Uses and Gratifications” theory. This classic framework in media use research focuses on the gratifications audiences derive from media content and the social-psychological variables influencing their media use. It posits that audiences engage with media in a goal-oriented manner, driven by personal needs that guide their interactions, during which their needs are fulfilled [53–54]. In the context of the Paris Olympics, the public satisfied their cognitive needs for event-related information by following media coverage, validating the theory`s applicability in the digital age`s media-public interaction dynamics.

Regional differences in media influence reveal the diversity of information environments: in areas with limited information channels, mainstream media play a prominent authoritative guiding role; whereas in regions with diversified information sources, the public is more likely to shift to other online information sources due to the weakened influence of mainstream media. This provides empirical evidence for understanding the relationship between media ecosystems and public attention allocation in the digital age.

From a practical perspective, the findings of this study can also assist media organizations in optimizing their reporting strategies, highlighting the appeal of events from the perspectives of hot topics and specific regions, thereby facilitating more effective event promotion and marketing strategies for event decision-makers and organizers. Additionally, these findings provide methodological references for enhancing media and public attention toward other large-scale events. In the context of digitalization and globalization, information dissemination channels have become diverse, and public information consumption habits are evolving. Media and public attention exhibit dynamic and complex characteristics. Future research should further explore how these factors influence the attention levels of large-scale sports events. Based on the current research findings, it is possible to predict future trends in sports event attention levels, propose corresponding strategic recommendations, and discuss how sports events can achieve balanced development in economic, social, and environmental benefits while enhancing attention levels.

### Copyrighted image

The authors declare that no copyrighted figures have been used in this manuscript. The base map (GS(2024)0650) was used in compliance with Chinese cartographic regulations. We overlaid analytical data using ArcGIS Pro.
